# Control of Neuropeptide Expression by Parallel Activity-dependent Pathways in *Caenorhabditis elegans*

**DOI:** 10.1038/srep38734

**Published:** 2017-01-31

**Authors:** Teresa Rojo Romanos, Jakob Gramstrup Petersen, Roger Pocock

**Affiliations:** 1 Development and Stem Cells Program, Monash Biomedicine Discovery Institute and Department of Anatomy and Developmental Biology, Monash University, Melbourne, Victoria 3800, Australia; 2 Biotech Research and Innovation Centre, University of Copenhagen, Ole Maaløes Vej 5, Copenhagen, Denmark

## Abstract

Monitoring of neuronal activity within circuits facilitates integrated responses and rapid changes in behavior. We have identified a system in *Caenorhabditis elegans* where neuropeptide expression is dependent on the ability of the BAG neurons to sense carbon dioxide. In *C. elegans,* CO_2_ sensing is predominantly coordinated by the BAG-expressed receptor-type guanylate cyclase GCY-9. GCY-9 binding to CO_2_ causes accumulation of cyclic GMP and opening of the cGMP-gated TAX-2/TAX-4 cation channels; provoking an integrated downstream cascade that enables *C. elegans* to avoid high CO_2_. Here we show that cGMP regulation by GCY-9 and the PDE-1 phosphodiesterase controls BAG expression of a FMRFamide-related neuropeptide FLP-19 reporter (*flp-19::GFP*). This regulation is specific for CO_2_-sensing function of the BAG neurons, as loss of oxygen sensing function does not affect *flp-19::GFP* expression. We also found that expression of *flp-19::GFP* is controlled in parallel to GCY-9 by the activity-dependent transcription factor CREB (CRH-1) and the cAMP-dependent protein kinase (KIN-2) signaling pathway. We therefore show that two parallel pathways regulate neuropeptide gene expression in the BAG sensory neurons: the ability to sense changes in carbon dioxide and CREB transcription factor. Such regulation may be required in particular environmental conditions to enable sophisticated behavioral decisions to be performed.

*C. elegans* performs multiple sensory modalities when navigating its environment. Such behaviors include sensing and responding to changes in temperature, atmospheric gases, food and mating^1^,^2^,^3^,^4^,^5^,^6^,^7^,^8^. Coordination of these complex behavioral responses requires constant monitoring of the status of the nervous system. Neuropeptide and neuropeptide receptor expression levels have previously been linked to the activity of neurons^4^,^9^,^10^,^11^,^12^,^13^. For example, insulin and neuropeptide signaling have been shown to report feeding status^14^. More recently, the regulation of expression of the serpentine receptor *srh-234* was linked with starvation, and dependent on the neuropeptide Y receptor NPR-1 and insulin-like growth factor receptor DAF-2^12^.

Despite having a small nervous system, the *C. elegans* genome encodes many neuropeptides^15^. These neuropeptides are classified into three main families: insulin-like family, neuropeptide-like protein family (NLPs) and FMRFamide-related peptides (FLPs). For FLP neuropeptides, the function for only a few are known suggesting that they work redundantly or are involved in fine-tuning of the nervous system in combination with neurotransmitter systems. However, certain functions have been elucidated for FLPs: FLP-11 has been identified as a regulator of a sleep-like state^16^, FLP-13 acts in the ALA neurons to regulate quiescence after heat-stress^17^, FLP-10 and FLP-17 inhibit egg laying^18^ and FLP-21 regulates social feeding behavior through the NPR-1 receptor^19^.

The *C. elegans* nervous system consists of 302 neurons, some of which function in non-overlapping circuits to regulate distinct behaviors. The BAG neurons for example are major regulators of acute CO_2_ avoidance behavior^5^,^6^, the regulation of pharyngeal pumping rate when animals are exposed to high levels of CO_2_^20^, and the control of egg laying^18^. The BAG neurons also act in conjunction with the URX neurons to coordinate behavioral responses to changes in O_2_ gradients. The BAG and URX neurons are activated when O_2_ levels drop and increase respectively^3^,^4^,^21^,^22^,^23^. However, the relationship between the BAG and URX neurons is not exclusive to O_2_ sensing: BAG-ablated animals live longer, while ablation of the URX neurons results in shorter lifespan, and these physiological changes are regulated by O_2_-sensing guanylate cyclases expressed in these neurons^24^. With such divergent and sophisticated roles for the BAG and URX neurons, prioritization and integration of information to guide behavioral responses may require modular regulation of neuropeptide expression.

Here we describe a system where the activity of the BAG neurons is crucial for the expression of a transcriptional reporter for the FLP-19 neuropeptide (*flp-19::GFP*). Furthermore, we show that the expression of *flp-19::GFP* is regulated via two parallel modules: 1) cGMP signaling through GCY-9 and PDE-1 and 2) the CREB transcription factor.

## Results

### *flp-19::GFP* Expression in the BAG Neurons Requires Cilia Function

It has previously been shown that neuropeptide expression is highly plastic, and can be controlled by neuronal activity^4^,^10^,^13^. The *flp-19::GFP* transcriptional reporter drives expression in the BAG, URX, AIN, AWA and HSN neurons in the hermaphrodite^25^. Our previous studies showed that *flp-19::GFP* expression in the BAG neurons is exquisitely sensitive to perturbations in parallel transcriptional pathways that control BAG cell fate and function^26^,^27^,^28^. We hypothesized that neuronal activity may underpin the transcriptional regulation of *flp-19::GFP* in the BAG neurons. We therefore tested if the expression of *flp-19::GFP* is compromised when the activity of the BAG neurons is reduced or abolished.

**Figure 1:**
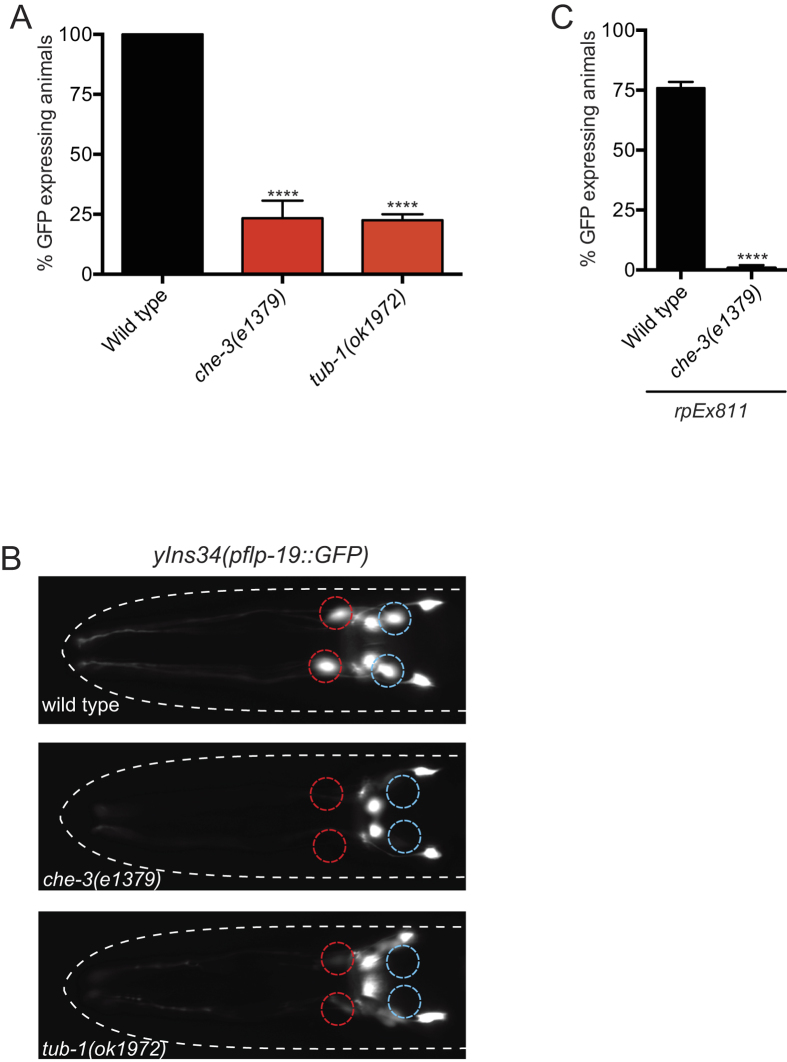
Cilia Function is Required for Expression *of flp-19::GFP* in the BAG Gas-sensing Neurons. (**A**) Quantification of the expression of the *flp-19::GFP* fluorescent reporter (*ynIs34*) in wild type, *che-3(e1379*) and *tub-1(ok1972*) mutant animals. n > 50. ****P < 0.0001. See Materials and Methods for neuronal scoring criteria used. (**B**) Micrographs show representative pictures of *flp-19::GFP(ynIs34*) expression in wild type, *che-3(e1379*) and *tub-1(ok1972*) strains. BAG positions are marked with red dashed circles. Note - expression of *flp-19::GFP* is also lost in the ciliated AWA neurons in *che-3(e1379*) and *tub-1(ok1972*) mutant animals (blue dotted circles). Anterior to the left. Scale bar = 20 μm. (**C**) Quantification of the expression of the *flp-19::GFP* reporter (*rpEx811*) in wild type and *che-3(e1379*) backgrounds. These data confirm the results shown in Fig. 1A. n > 50. ****P < 0.0001.

As the BAG neurons are ciliated, we first examined if disruption of cilia structure, which is required for several behaviors in *C. elegans*^7^,^29^,^30^, would affect *flp-19::GFP* expression. We crossed the *flp-19::GFP* reporter (*ynIs34*) with a mutant of *che-3*, which encodes a dynein that is required for structural integrity of sensory cilia. We observed that *che-3(e1379*) mutant animals exhibit strong defects in the expression of *flp-19::GFP* in the ciliated BAG and AWA neurons but not in the non-ciliated neurons (Fig. 1A,B). To verify this regulation, we crossed *che-3(e1379*) mutant animals with an independent *flp-19::GFP* reporter (*rpEx811*) and observed a similar phenotype (Fig. 1C). We next asked if disturbance of cilia transport would produce a similar effect on *flp-19::GFP* expression. TUB-1, homolog of TUBBY in mammals, is required for correct localization of G protein coupled receptors to cilia^31^,^32^,^33^. We found that removal of TUB-1 function phenocopied the *che-3* mutant loss of *flp-19::GFP* expression in the BAG neurons (Fig. 1A,B), indicating that correct cilia function is required for expression of *flp-19::GFP*.

### GCY-9 Regulates *flp-19::GFP* Expression Cell-autonomously in the BAG Neurons

**Figure 2:**
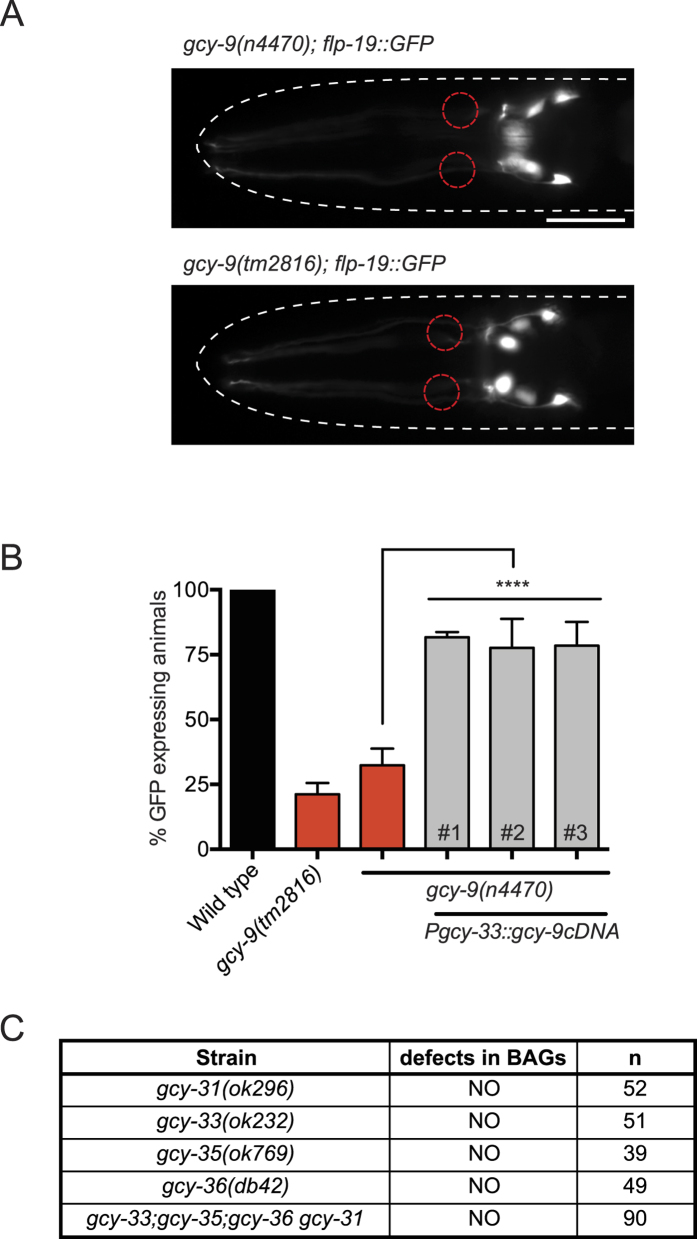
Autonomous CO_2_ Sensing is Required for *flp-19::GFP* Expression in the BAG Neurons. (**A**) *gcy-9(n4470*) and *gcy-9(tm2816*) mutants exhibit reduced expression of the *flp-19::GFP* reporter in the BAG neurons. Micrographs show representative images of *gcy-9* mutant hermaphrodites expressing the *flp-19::GFP* reporter (compare to Fig. 1B, top panel). BAG positions are marked with red dashed circles. Anterior to the left. Scale bar = 20 μm. (**B**) Quantification of *flp-19::GFP* expression in *gcy-9(n4470*) and *gcy-9(tm2816*) mutant animals. Transgenic expression of *gcy-9 cDNA* under the control of a BAG-specific *gcy-33* promoter rescues *gcy-9(n4470*) mutant phenotype. #1-3 indicate independent transgenic rescue lines. See Materials and Methods for neuronal scoring criteria used. n > 50. ****P < 0.0001. (**C**) Table showing the effect of loss of O_2_-sensing guanylate cyclases on *flp-19::GFP* expression in the BAG neurons. BAG guanylate cyclase functional nulls *gcy-31(ok296*) and *gcy-33(ok232*) do not exhibit defects in expression of *flp-19::GFP* in the BAG neurons. Mutants for URX guanylate cyclases *gcy-35(ok769*) and *gcy-36(db42*) do not exhibit defects in expression of *flp-19::GFP* in the BAG neurons. The quadruple *gcy-31 gcy-36; gcy-33; gcy-35* also does not exhibit any defects in BAG expression suggesting that *flp-19::GFP* expression in the BAG neurons is dependent on CO_2_ sensing and not O_2_ sensing.

Acute responses to CO_2_ are regulated by a neuronal circuit that includes the BAG neurons. The BAG neurons sense carbon dioxide through the GCY-9 receptor-type guanylate cyclase, homolog of the human GC-D^26^,^34^,^35^. As such, *gcy-9* mutants are unable to sense and respond to changes in CO_2_ concentration^34^. We asked whether the CO_2_-sensing function of the BAG neurons is required for *flp-19::GFP* expression. To this end, we crossed two independent deletion alleles of *gcy-9 (n4470, tm2816*) with the *flp-19::GFP* reporter and observed a reduced number of animals expressing GFP in the BAG neurons (Fig. 2A,B). When we resupplied *gcy-9 cDNA* driven by a BAG specific *gcy-33* promoter into *gcy-9(n4470*) mutant animals, *flp-19::GFP* expression was restored (Fig. 2B). The BAG neurons are also involved in sensing downshifts in O_2_ concentration, through expression of the soluble guanylate cyclases GCY-31 and GCY-33^22^. To ask whether O_2_-sensing function of the BAG neurons is also required to regulate *flp-19::GFP* expression, we crossed *gcy-31(ok296*) and *gcy-33(ok232*) mutants with the *flp-19::GFP* reporter. We found no detectable change in expression of *flp-19::GFP* when BAG O_2_-sensing function was ablated (Fig. 2C). As it has been previously shown that the URX and BAG communicate with each other^23^,^24^, we asked whether removal of O_2_-sensing from the URX neurons would affect the expression of *flp-19::GFP*. We therefore crossed the *flp-19::GFP* reporter into *gcy-35(ok769*) and *gcy-36(db42*) mutant strains, in which soluble guanylate cyclases required for URX O_2_-sensing function are mutated^3^,^4^,^22^,^36^. However, we observed no defect in the expression of *flp-19::GFP* in the BAG or URX neurons (Fig. 2C and data not shown). Furthermore, animals which lack O_2_ sensing function in both the BAG and URX neurons (*gcy-31 gcy-36; gcy-33; gcy-35* quadruple mutant) exhibit wild type expression of *flp-19::GFP* (Fig. 2C). Taken together, these data show that CO_2_ sensing function, and not O_2_ sensing function, regulates the expression of the *flp-19::GFP* reporter in the BAG neurons.

To ask whether CO_2_ sensing has a general effect on neuropeptide expression in the BAG neurons, we analyzed the expression of other neuropeptides in *gcy-9(n4470*) mutant animals. We crossed the *gcy-9(n4470*) mutant with reporters for *flp-17(ynIs64*) and *flp-13(ynIs37*) and observed no change of expression compared to wild type (Figure S1A). Furthermore we tested other reporters expressed in the BAG neurons (soluble guanylate cyclases *gcy-31(rpIs29*) and *gcy-33(rpIs7*) and the transcription factor *egl-13(rpIs32*)) in the *gcy-9* mutant background and we observed no change of expression (Figure S1A). Therefore, the regulation of *flp-19::GFP* expression by GCY-9 is somewhat specific.

### cGMP Levels Regulate *flp-19::GFP* Expression in the BAG Neurons

**Figure 3:**
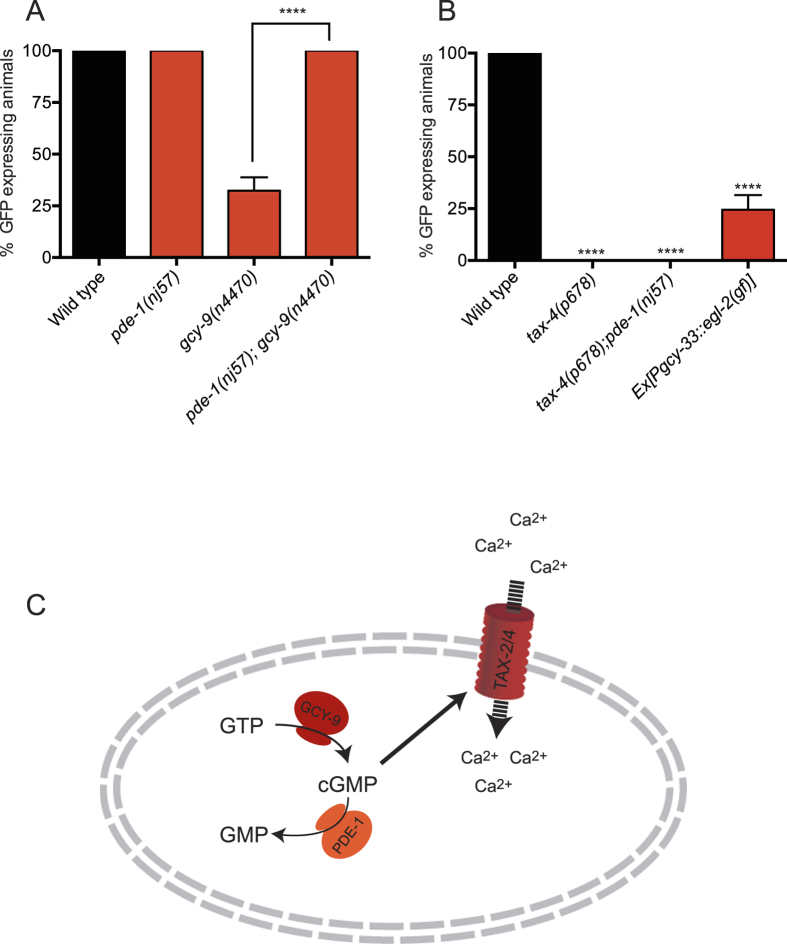
cGMP Levels Regulate *flp-19::GFP* Expression in the BAG Neurons. (**A**) Quantification of the *flp-19::GFP* reporter suggests that the guanylate cyclase GCY-9 and the phosphodiesterase PDE-1 exhibit opposing regulation of cGMP levels in the BAG neurons. The *pde-1(nj57*) mutant exhibits wild type expression of *flp-19::GFP* in the BAG neurons while *gcy-9(n4470*) mutants exhibit a strong defect in expression. In *gcy-9(n4470); pde-1(nj57*) double mutant animals, *flp-19::GFP* expression in the BAG neurons is restored, suggesting that cGMP levels in the BAG neurons regulate *flp-19::GFP* expression. See Materials and Methods for neuronal scoring criteria used. n > 50. ****P < 0.0001. (**B**) *flp-19::GFP* in the BAG neurons is completely abrogated in *tax-4(p678*) mutant animals. Furthermore, the *tax-4(p678*) mutant defect in *flp-19::GFP* expression cannot be rescued by mutation in *pde-1* indicating that *tax-4* acts downstream of cGMP signaling. Expression of an constitutively active form of EGL-2 in the BAGs also reduces *flp-19::GFP* expression indicating that *flp-19::GFP* expression is activity dependent. See Materials and Methods for neuronal scoring criteria used. n > 50. ****P < 0.0001. (**C**) Schematic model of cGMP regulation in the BAG neurons. GCY-9 converts GTP into cGMP, while PDE-1 catalyzes the conversion of cGMP into GMP. cGMP opens the TAX-2/TAX-4 channels leading to an influx of calcium and activation of the neuron.

*gcy-9* encodes a receptor-type guanylate cyclase. The role of these enzymes is to generate cGMP for gating downstream cyclic nucleotide-gated TAX-2/TAX-4 cation channels. As such GCY-9 regulates the activity of the BAG neurons through the control of cGMP levels. We speculated therefore that cGMP is a key regulator of *flp-19::GFP* expression in the BAG neurons. In order to test this hypothesis, we genetically manipulated cGMP levels. Phosphodiesterases are enzymes that catalyse the breakdown of cGMP to GMP and it has previously been shown that the phosphodiesterase PDE-1 is expressed in the BAG neurons^34^. We therefore asked whether the predicted increase in cGMP levels in *pde-1* mutant animals would affect *flp-19::GFP* expression. We crossed the *pde-1(nj57*) mutant with the *flp-19::GFP* reporter and observed that BAG expression of *flp-19::GFP* was unaffected (Fig. 3A). Next we tested if loss of *pde-1,* and therefore increase of cGMP in the BAG neurons, was sufficient to derepress *flp-19::GFP* expression in the *gcy-9* mutant. We therefore examined *flp-19::GFP* expression in the *pde-1(nj57); gcy-9(n4470*) double mutant and found that expression in the BAG neurons was fully restored (Fig. 3A). This suggests that reduced levels of cGMP in the BAG neurons causes transcriptional downregulation of *flp-19::GFP*.

Activation of GCY-9 by CO_2_ normally triggers the conversion of cGMP from GTP^35^. Subsequently, cGMP opens the cyclic nucleotide-gated channels TAX-2/TAX-4, through which the neuron is activated by calcium influx^5^,^6^. We therefore hypothesized that GCY-9-mediated regulation of the *flp-19::GFP* reporter was through this canonical pathway. Indeed, we found that in *tax-4(p678*) mutant animals *flp-19::GFP* expression is undetectable in the BAG neurons (Fig. 3B). In addition, we observed loss of *flp-19::GFP* expression in the URX O_2_-sensing neurons of *tax-4(p678*) mutant animals, suggesting that similar mechanisms of regulation may exist in these functionally-related neurons. However, we did not observe loss of *flp-19::GFP* expression in the *gcy-35* or *gcy-36* mutants or the quadruple *gcy-31 gcy-36; gcy-33; gcy-35* mutant strain (data not shown), suggesting that another pathway of regulation, independent of O_2_ sensing, regulates the expression of *flp-19::GFP* through the TAX-2/TAX-4 channels in the URX neurons. To confirm that the BAG neurons are present in *tax-4* mutant animals, we crossed *tax-4(p678*) mutant animals with an independent reporter for BAG neurons (*egl-13::GFP*), and observed no loss of expression (Figure S1B). Furthermore, we found that loss of *pde-1* was not able to restore the *tax-4(p678*) mutant *flp-19::GFP* expression, indicating that *tax-4* acts downstream of cGMP regulation, and that the channels are necessary for the expression of *flp-19::GFP* regardless of the levels of cGMP (Fig. 3B).

The data we have presented suggest that activity of the BAG neurons is important for the expression of *flp-19::GFP.* To reinforce this assertion, we inactivated the BAG neurons by expressing a constitutively-active EGL-2(GF) potassium channel using the *gcy-33* promoter^4^,^37^,^38^. We found that animals carrying the *Pgcy-33::egl-2(gf*) transgene exhibit a decrease in the expression of *flp-19::GFP* in the BAGs (Fig. 3B).

Taken together, our data show that GCY-9 and the downstream cGMP-regulated TAX-2/TAX-4 channels are required for the expression of *flp-19::GFP* in the BAG neurons. These data suggest that the conversion of GTP to cGMP by GCY-9 triggers opening of the TAX-2/TAX-4 channels, resulting in calcium influx and that this change in activity regulates the transcription of the neuropeptide reporter *flp-19::GFP* (Fig. 3C).

### *flp-19::GFP* Expression Does not Require Neuropeptide or Neurotransmitter Signaling

We have shown that the expression of *flp-19::GFP* is sensitive to GCY-9 and TAX-4-controlled activity. We next asked whether the expression of *flp-19::GFP* was regulated by neuropeptide and neurotransmitter signaling, through either an autocrine or paracrine fashion. We first tested whether *flp-19::GFP* expression was affected by abolishing neuropeptide signaling. To this end, we crossed the *flp-19::GFP* reporter with two mutants that do not have proper neuropeptide signaling: *egl-3(nr2090*), responsible for maturation of neuropeptides, and *unc-31(e169*), involved in dense core vesicle function^39^,^40^,^41^. The expression of *flp-19::GFP* in the BAG neurons in *egl-3* and *unc-31* mutants was unchanged when compared to wild type (Table 1). The BAG neurons are glutamatergic as they express the vesicular glutamate transporter EAT-4^42^,^43^. We found that the glutamatergic function of the BAG neurons is not required for the regulation of *flp-19::GFP* as expression is unchanged in an *eat-4(ky5*) mutant (Table 1). Furthermore, we asked if neurotransmitter release through synaptic vesicle exocytosis was required for *flp-19::GFP* expression. We crossed *flp-19::GFP* with the *unc-13(e1091*) mutant, defective in neurotransmission due to compromised synaptic vesicle fusion and the *snb-1(md247*) mutant, defective in synaptic transmission^44^,^45^,^46^. We found that *unc-13* and *snb-1* mutant animals exhibit wild type expression of *flp-19::GFP* in the BAG neurons (Table 1). These data suggest that the regulation of *flp-19::GFP* in the BAG neurons occurs through a BAG-intrinsic mechanism.

### CRH-1/CREB, an Activity-dependent Transcription Factor Acts in Parallel to GCY-9 to Regulate *flp-19::GFP* Expression in the BAG Neurons

**Figure 4:**
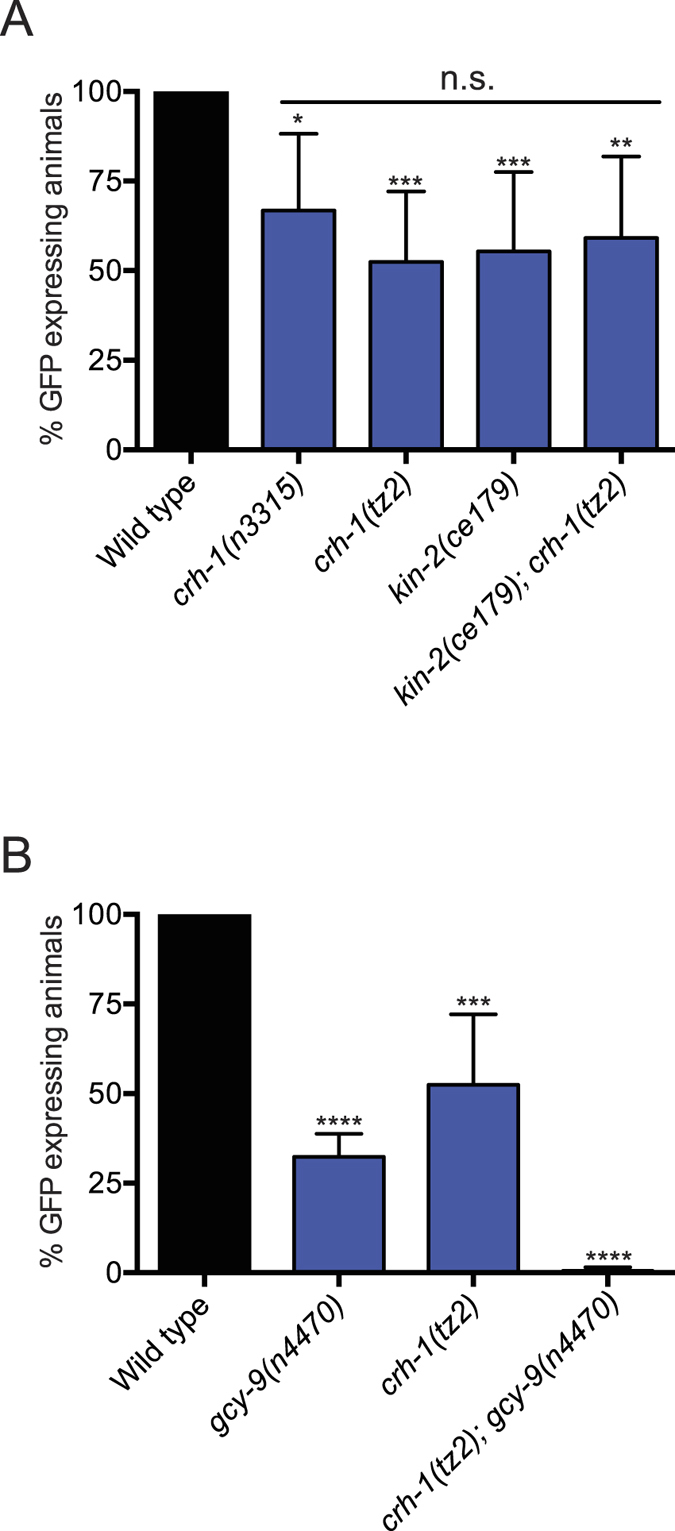
CREB/CRH-1 Acts in Parallel to GCY-9 to Regulate *flp-19::GFP* Expression in the BAG Neurons. (**A**) Quantification of the defects in *flp-19::GFP* expression in the BAG neurons in *crh-1* mutant alleles *tz2* and *n3315* and the *kin-2(ce179*) mutant. Both *crh-1* and *kin-2* mutants show a similar penetrance of defects in the expression of *flp-19::GFP* in the BAG neurons. Furthermore, the *kin-2(ce179); crh-1(tz2*) double mutant defect is not significantly different to either single mutant, suggesting that they act in the same genetic pathway. See Materials and Methods for neuronal scoring criteria used. n > 50. *P < 0.05, **P < 0.01, ****P < 0.0001, n.s. = not significantly different from wild type. (**B**) Quantification of the defects in *flp-19::GFP* expression in the BAG neurons in *gcy-9(n4470), crh-1(tz2*) and the *gcy-9(n4470); crh-1(tz2*) double mutant. While *crh-1* mutants show a partial defect in *flp-19::GFP* expression, in the *crh-1(tz2); gcy-9(n4470*) double mutant *flp-19::GFP* expression is undetectable. This indicates that *crh-1* acts independently to the *gcy-9* regulatory pathway. See Materials and Methods for neuronal scoring criteria used. n > 50. ***P < 0.001, ****P < 0.0001.

**Figure 5:**
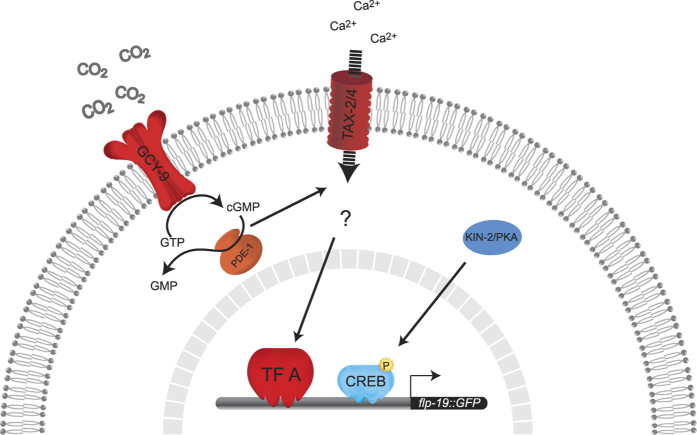
Expression of *flp-19::GFP* in the BAG Neurons is Controlled by Parallel Pathways. Schematic representation of the proposed model of regulation of *flp-19::GFP* expression in BAG neurons. The GCY-9 receptor binds CO_2_ converting GTP into cGMP. cGMP triggers opening of the cyclic nucleotide-gated TAX-2/TAX-4 cation channels, allowing Ca^2+^ to enter the neuron, leading to regulation of an unknown transcription factor (TF A) that positively regulates the expression of *flp-19::GFP*. PDE-1 reduces the levels of cGMP, opposing the action of GCY-9. In parallel, CRH-1 regulates the expression of *flp-19::GFP* by a signaling pathway involving the cAMP-dependent protein kinase KIN-2.

In order to better understand the mechanism through which *flp-19::GFP* is regulated, we studied mutants of various genes involved in activity-dependent expression in the nervous system (Table 1). Our screen found that both *crh-1* and *kin-2* mutants display defects in the expression of the *flp-19::GFP* reporter in the BAG neurons. CRH-1 is the *C. elegans* homolog of CREB and functions in neurons to regulate various behaviors. CRH-1 regulates lifespan, tap habituation and it has been linked with the control of thermotaxis behavior from the AFD neurons^47^,^48^,^49^. KIN-2 is the homolog of the regulatory subunit of cAMP-dependent protein kinase (PKA) and it can regulate CREB activity through a cascade of phosphorylation events^50^,^51^,^52^,^53^,^54^. We found that two independent null alleles of *crh-1 (tz2* and *n3315*) exhibit reduced expression of *flp-19::GFP* in the BAG neurons (Fig. 4A). In addition, *kin-2(ce179*) mutants show a similar phenotype to *crh-1* mutants, and the *crh-1(tz2); kin-2(ce179*) double mutant is not significantly different from either single mutant, suggesting that they function in the same genetic pathway (Fig. 4A). We speculated that CRH-1 might be the downstream effector in the GCY-9 cascade. To ask whether *gcy-9* and *crh-1* act in the same genetic pathway to control *flp-19::GFP* expression we constructed a *gcy-9(n4470); crh-1(tz2*) double mutant. Surprisingly, we found that these double mutant animals exhibited complete abrogation of *flp-19::GFP* expression (Fig. 4B). We confirmed that the BAG neurons are present in the *gcy-9(n4470); crh-1(tz2*) double mutant by examining a *flp-13::GFP* transgene (Figure S1C). Together, these data suggest that *gcy-9* and *crh-1* act in two separate genetic pathways to regulate *flp-19::*GFP expression (Fig. 5).

In summary, we propose a model where *flp-19::GFP* expression is regulated by two parallel pathways: 1) GCY-9 regulation of the TAX-2/TAX-4 cation channels through the control of cGMP levels, signaling to an unknown transcription factor and 2) CRH-1/CREB regulation by the kinase KIN-2. likely through a CO_2_-independent signaling pathway (Fig. 5).

## Discussion

Activity-dependent regulation is common occurrence in sensory neurons and can contribute in changes in behavior. Chemoreceptor genes can be regulated by different mechanisms such as developmental changes, neuronal activation, or in a paracrine fashion through pheromones. Transcriptional changes of chemoreceptors might be a strategy to modulate external responses. For example, the TAX-2/TAX-4 calcium channel regulates the expression of chemoreceptors such as STR-2 and SRD-1 in the AWC and ASI neurons respectively^11^,^55^. In addition, TRPV channels control the biosynthesis of serotonin through regulation of tryptophan hydroxylase expression in the ADF neurons^56^.

We have presented data to show that *flp-19::GFP* expression in the BAG neurons is regulated by two parallel pathways. The GCY-9 pathway controls *flp-19::GFP* expression by modulating the levels of cGMP, counterbalanced by the phosphodiesterase PDE-1. PDEs and GCYs are known to function together in other neurons to regulate activity. For example, *gcy-12* and *pde-2* control cGMP levels to determine body size in *C. elegans*^57^. Similarly, in the AFD neurons, the opposing roles of GCY-8 and PDE-2, control *C. elegans* thermotaxis behavior^58^. It might be possible that PDE-1 is involved in sensing the levels of CO_2_ in the BAG neurons; potentially to set a quantitative threshold or temporal window of TAX-2/TAX-4 channel opening. We have not identified the effector downstream the TAX-2/TAX-4 signaling, but we speculate that it may be a transcription factor regulated by calcium or calmodulin dependent activation. In parallel to GCY-9, CRH-1/CREB also controls the level of *flp-19::GFP* through the activity of the PKA kinase. As PKA is a cAMP regulated kinase, this may suggest that a cAMP-regulated pathway controls *flp-19::GFP* expression through CRH-1.

It is necessary to further study the implications of the two parallel pathways we have identified to understand the advantages they may provide in the natural habitat of *C. elegans*. Interestingly worms are attracted to CO_2_ as dauers while L4 larvae avoid CO_2_^5^,^6^,^34^. Furthermore it has been shown how in juvenile infective stages of parasitic worms (related to *C. elegans*) BAG neurons are involved in the attraction to CO_2_^59^. It might be possible that this change in the attraction/repulsion to CO_2_ is regulated by the expression of neuropeptides such as FLP-19. When worms are grown in low O_2_ levels, they become attracted to lower levels of O_2_, instead of being repelled. This change of behavior is due to changes in the expression level of guanylate cyclases that detect O_2_. It might be possible that similar adaptation occurs when worms are grown in hypercapnic conditions, and the transcriptional regulation of *flp-19* could be involved in such adaptation. However, the role of *flp-19* may not be directly related to CO_2_ sensing, but to other broader functions. Indeed, the BAG neurons are involved in lifespan regulation, as is the CRH-1 transcription factor, therefore it would be interesting to examine whether *flp-19* mutant animals display defects in longevity.

*flp-19* is expressed in a subset of neurons that are distinct in class and function: from oxygen (URX/BAG) and carbon dioxide sensing (BAG), chemotaxis (AWA), egg laying (HSN) or pheromone sensing (CEM). Similar means of *flp-19* activity-dependent regulation maybe present in these other neurons through different molecular pathways, providing this neuropeptide with multiple layers of control that may be required in particular ephemeral habitats.

## Methods

### Strains used in this study

Strains were grown using standard growth conditions on NGM agar at 20 °C on *Escherichia coli* OP50^60^. Transgenic animals were created as previously described^61^. Strain information is detailed in Table S1.

### Constructs and generation of transgenic worms

The *Pgcy-33::gcy-9 cDNA* rescue construct was generated by cloning the 1 kb *gcy-33* promoter using HindIII and BamHI and *gcy-9cDNA,* using KpnI and NheI into pPD49.26 vector into multiple cloning sites 1 and 2 respectively (Fire Vector Kit). Transgenic animals were obtained through microinjection^61^. The construct was injected into young adult hermaphrodites as a simple array (20 ng/ul *Pgcy-33::gcy-9 cDNA*, 10 ng/ul *myo-3::RFP* as co-injection marker).

The *BAG::egl-2(gf*) construct was generated by cloning the 1 kb *gcy-33* promoter using HindIII and BamHI and *egl-2(gf)cDNA* (kind gift of Mario De Bono) using KpnI into the pPD49.26 vector into multiple cloning sites 1 and 2 respectively (Fire Vector Kit). The construct was injected as simple array (50 ng/ul *egl-2(gf*) and 5 ng/ul myo-3::RFP as co-injection marker).

### Microscopy

Worms were anesthetized in 20 mM NaN_3_ on 5% agarose on glass slides and images were taken using an upright fluorescence microscope (Zeiss, AXIO Imager M2) and ZEN software (version 2.0). Neuronal scoring: Neurons were given a numerical value according to their expression levels. Wild-type expression scored 1, decreased expression scored 0.5 and abolished expression scored 0. Percentage of GFP expressing animals was then correlated to the theoretical maximum score using the equation below.


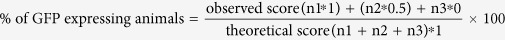


### Statistical analysis

Statistical analysis was performed in GraphPad Prism 6 using one-way ANOVA with Newman-Keuls Multiple Comparison Test. Values are expressed as mean +/− s.d. Differences with a P value < 0.05 were considered significant.

## Additional Information

**How to cite this article**: Romanos, T. R. *et al*. Control of Neuropeptide Expression by Parallel Activity-dependent Pathways in *Caenorhabditis elegans. Sci. Rep.* **7**, 38734; doi: 10.1038/srep38734 (2017).

**Publisher's note:** Springer Nature remains neutral with regard to jurisdictional claims in published maps and institutional affiliations.

## Supplementary Material

Supplementary Information

## Figures and Tables

**Table 1:** Genes Tested for Expression of *flp-19::GFP* in the BAG Neurons.

Strain	Defects in BAGs	n
**Neuropeptide**		
*egl-3(nr2090*)	NO	62
*unc-31(e169*)	NO	56
**Neurotransmitter**		
*unc-13(e1091*)	NO	51
*eat-4(ky5*)	NO	58
*snb-1(md247*)	NO	67
**Activity-related genes**		
*crh-1(tz2*)	YES	111
*crh-1(n3315*)	YES	75
*kin-2(ce179*)	YES	193
*jun-1(gk557*)	NO	90
*egl-4(n479*)	NO	41
*cmk-1(ok287*)	NO	58
*ckk-1(ok1033*)	NO	51
*rgs-3(ok2288*)	NO	51

We crossed the *flp-19::GFP* reporter with mutants for neuropeptide function, neurotransmission and synaptic regulators and other activity-related genes. From the screen, we found that *crh-1* and *kin-2* are necessary for the proper expression of the *flp-19::GFP* reporter.
