# Supplementary material for: Control of Neuropeptide Expression by Parallel Activity-dependent Pathways in Caenorhabditis elegans
Source: Sci Rep. 2017 Jan 31;7:38734. doi: 10.1038/srep38734 (PMC5282578; doi:10.1038/srep38734)
Supplement: Supplementary Information [file srep38734-s1.pdf]

Control of Neuropeptide Expression by Parallel Activity-dependent Pathways in *Caenorhabditis elegans*

Teresa Rojo Romanos[1,2], Jakob Gramstrup Petersen[2] and Roger Pocock[1,2]

Figure S1.

(A) Table showing the BAG reporters tested for expression in the *gcy-9(n4470)* mutant. None of the reporters exhibited a detectable defect in BAG expression.

(B) The BAG neurons are generated in *tax-4(p678)* mutant animals. Representative micrograph showing the expression of the BAG reporter *rpIs32(Pegl-13::GFP)* in a hermaphrodite worm mutant for *tax-4(p678)*. Both BAG neurons show wild type expression of the reporter. BAG positions are marked with red dashed circles. Anterior to the left. Scale bar = 20μm.

(C) Micrograph showing the expression of *flp-13::GFP* in the *crh-1(tz2); gcy-9(n4470)* double mutant. Expression of the reporter in is unaffected in the BAG neurons. BAG positions are marked with red dashed circles. Anterior to the left. Scale bar = 20μm.

Table S1.

List of strains used in this study.

A

gcy-9(n4470)		
**Transgene**	**Defective**	**n**
ynIs64[flp-17::GFP]	NO	55
ynIs37[flp-13::GFP]	NO	54
rpIs29[gcy-31::mCherry]	NO	69
rpIs7[gcy-33::GFP]	NO	51
rpIs32[egl-13::GFP]	NO	61

B

tax-4(p678); egl-13::GFP

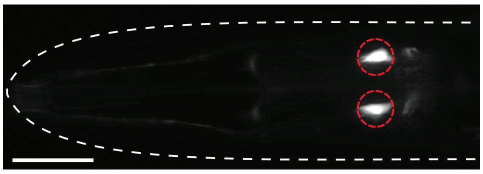

C

crh-1(tz2); gcy-9(n4470); flp-13::GFP

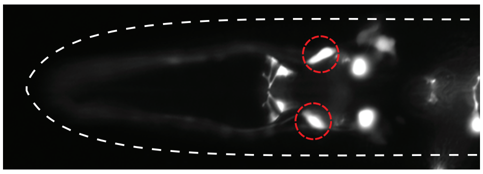

Figure S1

	Strain number	Genotype
**Figure 1**	RJP255	*ynIs34[flp-19::gfp]; him-5(e1490)*
	RJP1206	*che-3(e1379); ynIs34[flp-19::gfp]*
	RJP1143	*tub-1 (ok1972); ynIs34[flp-19::gfp]*
	RJP1968	*rpEx811[flp-19::GFP]*
	RJP1969	*che-3(e1379); rpEx811[flp-19::GFP]*
**Figure 2**	RJP1254	*gcy-9(n4470); ynIs34[flp-19::gfp]*
	RJP1560	*gcy-9(tm2816); ynIs34[flp-19::gfp]*
	RJP1814	*gcy-9(n4470); ynIs34[flp-19::gfp]; rpEx765[gcy-331K::gcy-9cDNA + myo-3::RFP]*
	RJP1815	*gcy-9(n4470); ynIs34[flp-19::gfp]; rpEx766[gcy-331K::gcy-9cDNA + myo-3::RFP]*
	RJP2027	*gcy-9(n4470); ynIs34[flp-19::gfp]; rpEx837[gcy-331K::gcy-9cDNA + myo-3::RFP]*
	RJP1268	*gcy-31(ok296); ynIs34[flp-19::gfp]; him-5(e1490)*
	RJP1247	*gcy-33(ok232); ynIs34[flp-19::gfp]*
	RJP1459	*gcy-35(ok769); ynIs34[flp-19::gfp]; him-5(e1490)*
	RJP1260	*gcy-36(db42); ynIs34[flp-19::gfp]*
	RJP3000	*gcy-35(ok769); gcy-33(ok232); gcy-36(db42) gcy-31(ok296); ynIs34[flp-19::gfp]*
**Figure 3**	RJP3008	*pde-1(nj57); ynIs34[flp-19::gfp]*
	RJP3033	*pde-1(nj57); gcy-9(n4470); ynIs34[flp-19::gfp]*
	RJP1388	*tax-4(p678); ynIs34[flp-19::gfp]*
	RJP2021	*tax-4(p678); pde-1(nj57); ynIs34[flp-19::gfp]*
	RJP3226	*ynIs34[flp-19::gfp]; rpEx1563[gcy-33::egl-2(gf) + myo-3::rfp]*
**Figure 4**	RJP3293	*crh-1(n3315); ynIs34[flp-19::gfp]*
	RJP1365	*crh-1(tz2); ynIs34[flp-19::gfp]; him-5(e1490)*
	RJP3031	*kin-2(ce179); ynIs34[flp-19::gfp]*
	RJP3277	*crh-1(tz2); kin-2(e179); ynIs34[flp-19::gfp]*
	RJP3032	*crh-1(tz2); gcy-9(n4470); ynIs34[flp-19::gfp]*
**Suppl Fig 1**	RJP1387	*gcy-9(n4470); ynIs64[flp-17::GFP]*
	RJP1566	*gcy-9(n4470); ylnIs37[flp-13::GFP]*
	RJP1567	*gcy-9(n4470); rpIs29[gcy-31::mCherry; elt-2::gfp]*
	RJP1558	*gcy-9(n4470); rpIs7[gcy-33::GCY-33::GFP]*
	RJP1534	*gcy-9(n4470); rpIs32[egl-13::GFP]*
	RJP1574	*tax-4(p678); rpIs32[egl-13::GFP]*
	RJP3123	*crh-1(tz2); gcy-9(n4470); ynIs37[flp-13::GFP]*
**Table 1**	RJP1792	*egl-3(nr2090); ynIs34[flp-19::gfp]*
	RJP1953	*unc-31(e169); ynIs34[flp-19::gfp]*
	RJP1453	*unc-13(e1091); ynIs34[flp-19::gfp]*
	RJP1580	*eat-4(ky5); ynIs34[flp-19::gfp]*
	RJP1796	*snb-1(md247); ynIs34[flp-19::gfp]; him-5(e1490)*
	RJP3273	*jun-1(gk557); ynIs34[flp-19::gfp]*
	RJP1967	*egl-4(n479); ynIs34[flp-19::gfp]*
	RJP1570	*cmk-1(ok287); ynIs34[flp-19::gfp]*
	RJP1556	*ckk-1(ok1033); ynIs34[flp-19::gfp]; him-5(e1490)*
	RJP1370	*rgs-3(ok2288); ynIs34[flp-19::gfp]*

Table S1
